# Intravitreal Injection of Bevacizumab: Review of our previous Experience

**Published:** 2018

**Authors:** Mehrdad Afarid, Ali Sadegi Sarvestani, Feisal Rahat, Ali Azimi

**Affiliations:** *Poostchi Ophthalmology Research Center, Shiraz University of Medical Sciences ,Shiraz,Iran.*

**Keywords:** Bevacizumab, Intravitreal Injection, Endophthalmitis, Review Article

## Abstract

Several ocular and systemic complications have been reported after the bevacizumab intravitreal injection. This study aims at reporting the main indications for the bevacizumab intravitreal injection in our center, the intravitreal injection method in this study, and the incidence of the post-injection complication, such as endophthalmitis.This study is a retrospective review of the consecutive intravitreal bevacizumab (Avastin®) injections for 359 patients between 2011 and 2013 at a single institute (Poostchi Clinic of Ophthalmology). Before the injection, a drop containing 5 mLCiprofloxacin and 5 mL Betadine 10% was applied 3 times at the intervals of 10 min. The eye lashes, upper and lower eyelids, and caruncle were swabbed with Betadine 10% but the lid speculum, drape, and conjunctival washing were not conducted.The patients were followed up 8 weeks after the injection for the evaluation of any complications.In this study, 1376 intravitreal injection of bevacizumab in 479 eyes of 359 patients were enrolled. Among them, 141patients (39.3%) were men and 218 (60.7%) were women. The mean age ( ± SD) of the patients was 61.48 (± 11.21) years. On average, each patient received 3.83 (the range 1-13) injections. The most common indications for the bevacizumab intravitreal injection were diabetic retinopathy, choroidal neovascularization, and central retinal vein occlusion. None of the patients developed endophthalmitis, retinal detachment, or other adverse effects. This study showed that the above-mentioned method of the intravitreal bevacizumab injection is easy and safe. The future studies involving more participants are required for the evaluation of rare complications.

## Introduction

Intravitreal injections (IVI) play a major role in the routine ophthalmic practice. The intravitreal injection is carried out for many purposes, and numerous medications, including antibiotics, antivirals, antifungals, corticosteroids, and anti-vascular endothelial growth factor (VEGF) agents are utilized for these purposes (1).

The level of VEGF in the ocular tissue is higher in some conditions, such as diabetic retinopathy, venous occlusive disorders, and age-related macular degeneration (2).One of the new treatments for these conditions is to use the intravitreal injection of the anti-VEGF agents like bevacizumab (Avastin®) (3).

Bevacizumab is a recombinant, full-length, humanized antibody that binds all VEGF isoforms (4). Avastin was used for the treatment of colorectal cancers in 2004 (5). Then the administration of Avastin became popular among specialists, including ophthalmologists for different diseases, such as: choroidal neovascularization (CNV) secondary to pathologic myopia (6) or secondary to age-related macular degeneration (AMD) (7), proliferative diabetic retinopathy (PDR) (8), diabetic macular edema (DME) (9), retinopathy of prematurity (10), iris neovascularization, and neovascular glaucoma (NVG) (11), and macular edema due to branch and central retinal vein occlusion (BRVO and CRVO) (12). Yet using the intravitreal bevacizumab is off-label for these conditions (13).

Several ocular and systemic complications have been reported after the bevacizumab intravitreal injection. Ocular side effects like the subconjunctival hemorrhage, transient and permanent rise of intraocular pressure, uveitis, increase the risk of the cataract, conjunctival chemosis, iatrogenic vitreous hemorrhage, rhegmatogenous retinal detachment, progression of diabetic tractional retinal detachment, sterile endophthalmitis, and finally infectious endophthalmitis as the most catastrophic complication (14-16) .

The most common systemic complications are the acute rise of blood pressure, mild irritation and allergic dermal reaction, myocardial infarction, stroke, and even death (14, 17).

Due to the importance of infectious endophthalmitis, which is one of the sight-threatening conditions in ophthalmology, surgeons try to use different methods for intravitreal injections to decrease the risk of the mentioned problem. Hence, in this study we seek to report our method of the bevacizumab intravitreal injection for different ocular diseases and the rate of the post-injection endophthalmitis, as well as the main indications for the bevacizumab intravitreal injection, highly beneficial for health policy makers. 

## Experimental

This study is a retrospective review of the consecutive intravitreal bevacizumab injections for 359 patients between 2011 and 2013 at a single institute (Poostchi Clinic of Ophthalmology).The medical records of 359 patients who had undergone the intravitreal injection by a single surgeon were reviewed for any documented complications, which include, yet are not limited to endophthalmitis, vitreous hemorrhage, traumatic cataract, and retinal detachment. Our method of injection was:

After complete ophthalmologic examinations, the informed consent was received from each patient before each injection. 

**Table 1:** Demographic characteristic of patients receiving intravitreal Bevacizumab (Avastin).

**Demographic characteristic**	
Number of patients	359
Mean of Age (Range)	61.48 (22-87)
Men (n) (%)	141 (39.27)
Women (n) (%)	218 (60.72)
Number of injections (mean for each eye)	1376 (3.83)
Right eye	249
Left eye	269
Diabetic Mellitus (n) (%)	275 (76.60)

**Table 2:** Indication for injection of Bevacizumab (Avastin).

**Indication**	**Number of patients**
CSME	221 (61.55%)
PDR with VH	23 (6.40%)
CNV	59 (16.43%)
CRVO	28 (7.79%)
BRVO	23 (6.40%)
NVG	4 (1.11%)
Parafoveal Telangiectasia	1 (0.27%)

CSME = Clinically Significant Macular Edema, PDR = Proliferative Diabetic Retinopathy, CNV = Choroidal neovascularization, CRVO = Central Retinal Vein Occlusion, BRVO = Branch Retinal Vein Occlusion, NVG = Neovascular Glaucoma

All injections were performed in an operating room. Bevacizumab (Avastin®; Genentech Inc., San Francisco, CA, USA) 1.25 mg/0.05 mL was aspirated with a 30-gauge needle from each vial (100mg/4mL) with a maximum of 40 consecutive injections aspirated in the operating room from the same vial. The utilized vials were then discarded without any overnight storage.

After the topical anesthesia (tetracaine), a drop consisting of 5 mL Ciprofloxacin and 5 mL Betadine10% (Ciprofloxacin and Betadine 5%) was applied 3 times at the intervals of 10 min to the affected eye. The oral acetazolamide (250 mg) was used by patients 30 min before the injection. The surgeon used the sterile gloves and surgical face mask, and the patients made use of the surgical gowns and surgical face masks. The surrounding eyelashes, upper and lower eyelids and caruncle were swabbed with Betadine 10%. The lid speculum, drape and conjunctival washing were not carried out. The 30-gauge needles were used. While the patient was asked to look down, by a sterile Gauze the upper lid was elevated, the superior bulbar conjunctiva was exposed and the eyelashes were completely covered. 

In pseudophakic or phakic patients, the injection site was 3.5–3.75 mm posterior to the limbus supratemporally, and the needle was directed toward the center of the vitreous cavity.

Each patient was given the Ciprofloxacin eye drop to be instilled hourly for the first 24 h and 4 times a day for 5 days. Each patient was given the written post-operative instructions and forewarned of the alarming signs, such as the ocular pain, decreased vision, and lid edema. One day after injection, the patients were evaluated for any sign of endophthalmitis and other complications.

After 24 h, the patients were evaluated for the conjunctival injection, anterior chamber reaction (ACR) or vitritis, and those with ACR were re-evaluated after 12-24 h. Any patient with significant (> 2 +) vitritis or hypopion was considered as a case of endophthalmitis. The patients were followed up 8 weeks after the injection for the evaluation of any complications.

The SPSS software version 15.0 was used to analyze the data.

## Results

In this study, 1376 intravitreal injection of bevacizumab (Avastin®) in 479 eyes of 359 patients were enrolled. Of these patients, 141(39.3%) were men and 218 (60.7%) were women. The demographic characteristics of the patients have been summarized in Table 1. The mean age (± SD) of the patients was 61.48 (± 11.21) years. On average, each patient received 3.83 (the range 1-13) injections.

The most common indications for the bevacizumab intravitreal injection were the diabetic retinopathy (clinically significant macular edema and proliferative diabetic retinopathy with vitreous hemorrhage), choroidal neovascularization, and CRVO. The indications for the bevacizumab intravitreal injections have been summed up in Table 2.

None of the patients developed endophthalmitis, retinal detachment, or other adverse effects.

## Discussion

The anti-vascular endothelial growth factors (Anti-VEGFs) change the treatment pattern of some ocular diseases.The management of the wet AMD, diabetic macular edema, CRVO, and BRVO has been improved by anti-VEGFs. Anti-VEGFs have been approved for the treatment of certain ocular diseases but the intravitreal injection of bevacizumab is off-label. Bevacizumab (Avastin®) is a non-selective antibody, which binds to all the VEGF isoforms. Because of the economic factors, the use of bevacizumab has increased and many ophthalmologists have used it as a first-line treatment in many ocular neovascular diseases. 

The ocular and systemic side effects of Anti-VEGFs were addressed by a number of studies (14-18). The safety issues of bevacizumab were reported in some other studies (19). The endophthalmitis, elevated intra-ocular pressure, subconjunctival hemorrhage, sterile uveitis, stroke, and myocardial infarction are some of the reported complications after the intravitreal injection of bevacizumab (14, 15).

One of the main catastrophic complications of the bevacizumab intravitreal injection is endophthalmitis. Different reports are present about the incidence of the post-injection endophthalmitis (20-22). The surgical method and pre- and post-operation medications are the factors that may affect the rate of endophthalmitis in these reports.

The results of our study showed that by use of prophylactic Betadine and antibiotic, the incidence of the post-intravitreal injection endophthalmitis can be decreased. However, our study is not a randomized controlled clinical trial and it is merely a report from one surgical method on behalf of a single surgeon. 

In most reports the betadine solution has been used before injection but the use of antibiotics before or after injection has been controversial in such reports (23, 24). Some studies reported patients with endophthalmitis, who were not given topical antibiotics before or after injection (25), but the role of the topical antibiotics for the prophylaxis of endophthalmitis after the intravitreal injections has not been proved in robust studies (26).

The use of the sterile gloves or sterile drapes is also controversial (23). We used the sterile gloves and surgical face mask but did not use the drape because the patient could look down more easily during the procedure. We also did not use the speculum for injections and we opened the eyelid with sterile gauze because the placement of a lid speculum before the intravitreal injection can be a highly painful procedure for a patient and decrease his/her compliance during the procedure. Some studies reported that the use of the eye speculum can be preventive for endophthalmitis (27) but it has yet to be proved in a randomized controlled clinical trial and the use of the eye speculum is controversial (28). On the other hand, preparing many sterile speculums for multiple injections in a single day may be difficult for small clinics; thus, we used sterile gauze to open the eyelids and cover the eyelashes.

Washing the conjunctiva with the balanced salt solution or normal saline for the prevention of endophthalmitis has also been argumentative in different studies (29) but its role is yet to be confirmed. Also, we did not wash the conjunctiva with fluid. 

The hemisphere of the injection (superior vs. inferior) has also been mentioned as a risk factor. Some studies reported that the superior injection is associated with the lower rate of endophthalmitis, yet this has not been confirmed in robust studies (30). We made the injections at the superior part of glob in nearly all the patients. Had a retinal break taken place at the superior retina after injection, its management would have become easier with the pneumatic retinopexy. 

The evaluation of the lid margin is a rule in all intraocular surgeries and we did not administer the injections to an eye with any sign of blepharitis.

Because Avastin is used as a 100/4 (mg/cc) vial and applied for many patients, the vial aspiration and handling method is a highly crucial factor. We aspirated all sterile syringes in one session in an operating room and then threw away the vial. We did not reuse the vials for the other injection sessions on the other days.

In the previous studies no significant differences were present between different anti-VEGFs in terms of endophthalmitis (30) but due to the economic factors and the lower cost of bevacizumab in comparison with ranibizumab or aflibercept, the use of bevacizumab has increased in recent years.

The intravitreal injections in an operating room setting were associated with a 13-fold lower risk of endophthalmitis, as compared with the in-office injections. Therefore, we made all the injections in an operating room setting (31

Like some other studies, the main reasons for injection in our study were diabetic retinopathy, retinal vascular accidents, and AMD.

As the injection method is an important factor for the development of endophthalmitis, it must be investigated in order to find the safest and easiest method, and if possible, each clinic or hospital must standardize the method of the bevacizumab intravitreal injection according to its facilities by reviewing different methods.

The importance of our study is to report a method of injection and at least it can be stated that the rate of endophthalmitis is no more than that in the previous reports, but more injections by this method should be carried out to better evaluate the rate of endophthalmitis.

## Conclusion

This study showed that the above-mentioned method of the intravitreal bevacizumab injection is easy and safe. The future studies involving more participants are required for the evaluation of rare complications, such as endophthalmitis and retinal detachment.
